# Chronic hM4Di-DREADD-Mediated Chemogenetic Inhibition of Forebrain Excitatory Neurons in Postnatal or Juvenile Life Does Not Alter Adult Mood-Related Behavior

**DOI:** 10.1523/ENEURO.0381-21.2021

**Published:** 2022-02-14

**Authors:** Praachi Tiwari, Darshana Kapri, Amartya Pradhan, Angarika Balakrishnan, Pratik R. Chaudhari, Vidita A. Vaidya

**Affiliations:** Department of Biological Sciences, Tata Institute of Fundamental Research, Mumbai 400005, India

**Keywords:** anxiety, depression, DREADD, early life, G_i_ signaling, schizophrenia

## Abstract

G-protein-coupled receptors (GPCRs) coupled to G_i_ signaling, in particular downstream of monoaminergic neurotransmission, are posited to play a key role during developmental epochs (postnatal and juvenile) in shaping the emergence of adult anxiodepressive behaviors and sensorimotor gating. To address the role of G_i_ signaling in these developmental windows, we used a CaMKIIα-tTA::TRE hM4Di bigenic mouse line to express the hM4Di-DREADD (designer receptor exclusively activated by designer drugs) in forebrain excitatory neurons and enhanced G_i_ signaling via chronic administration of the DREADD agonist, clozapine-*N*-oxide (CNO) in the postnatal window (postnatal days 2–14) or the juvenile window (postnatal days 28–40). We confirmed that the expression of the HA-tagged hM4Di-DREADD was restricted to CaMKIIα-positive neurons in the forebrain, and that the administration of CNO in postnatal or juvenile windows evoked inhibition in forebrain circuits of the hippocampus and cortex, as indicated by a decline in expression of the neuronal activity marker c-Fos. hM4Di-DREADD-mediated inhibition of CaMKIIα-positive forebrain excitatory neurons in postnatal or juvenile life did not impact the weight profile of mouse pups, and also did not influence the normal ontogeny of sensory reflexes. Further, postnatal or juvenile hM4Di-DREADD-mediated inhibition of CaMKIIα-positive forebrain excitatory neurons did not alter anxiety- or despair-like behaviors in adulthood and did not impact sensorimotor gating. Collectively, these results indicate that chemogenetic induction of G_i_ signaling in CaMKIIα-positive forebrain excitatory neurons in postnatal and juvenile temporal windows does not appear to impinge on the programming of anxiodepressive behaviors in adulthood.

## Significance Statement

The experience of early adversity can program persistent alterations in mood-states. It has been suggested that a perturbation of signaling pathways within forebrain neurocircuits, in particular a disruption of the balance between G_q_ and G_i_ signaling in forebrain excitatory neurons during critical developmental epochs may program the dysregulation of anxiodepressive behaviors. Prior evidence indicates that increased G_q_ signaling-mediated activation of forebrain excitatory neurons in postnatal life can enhance adult anxiodepressive behaviors. Here, we have addressed whether G_i_ signaling-mediated inhibition of forebrain excitatory neurons in the postnatal and juvenile windows of life can influence adult anxiodepressive behaviors. Our findings indicate that chronic chemogenetic inhibition of forebrain excitatory neurons via G_i_-mediated signaling during critical developmental time windows does not impact mood-related behavior.

## Introduction

Experiences during early developmental windows play a crucial role in the fine-tuning and shaping of an individual’s behavioral and functional responses in adulthood (Gross and Hen, 2004; Hensch, 2005; Ansorge et al., 2007; Bale et al., 2010; Di Segni et al., 2018). While exposure to early stress and trauma is associated with persistent increases in anxiety- and despair-like behavior in preclinical studies (Chen and Baram, 2016; De Melo et al., 2018; Targum and Nemeroff, 2019; Wang et al., 2020), enriched environment exposure (Kempermann et al., 1997; Francis et al., 2002; Cymerblit-Sabba et al., 2013; Ravenelle et al., 2014; Sparling et al., 2018) and high maternal care during these early temporal windows is associated with enhanced stress coping and resilient behavioral responses (Bredy et al., 2003; Champagne et al., 2008; Bagot et al., 2009). The neurotransmitter, 5-hydroxytryptamine (serotonin; 5-HT), and signaling via the G_i_-coupled 5-HT_1A_ and G_q_-coupled 5-HT_2A_ receptors, has been implicated in playing an important role in shaping the development of mood-related behavior (Gordon and Hen, 2004; Altieri et al., 2015; Tiwari et al., 2021). Elevation of 5-HT levels during postnatal life, either via pharmacological blockade or genetic loss of function of the 5-HT transporter, is associated with enhanced anxiety- and despair-like behavior that persists across the life span (Ansorge et al., 2004, 2008; Sarkar et al., 2014). Loss of function of the G_q_-coupled 5-HT_2A_ receptor, in particular in the forebrain, is associated with reduced anxiety-like behavior (Weisstaub, 2006), whereas loss of function of the G_i_-coupled 5-HT_1A_ receptor during postnatal life, in both forebrain and raphe neurocircuits, has been linked to increased anxiety-like behavior (Gross et al., 2002; Vinkers et al., 2010; Richardson-Jones et al., 2010, 2011; Mineur et al., 2015). Furthermore, pharmacological blockade of the G_i_-coupled 5-HT_1A_ receptor during postnatal life is associated with the emergence of increased anxiety in adulthood (Vinkers et al., 2010; Garcia-Garcia et al., 2014; Sarkar et al., 2014), whereas pharmacological stimulation of the G_q_-coupled 5-HT_2A_ receptors (Sarkar et al., 2014) or enhanced G_q_ signaling driven via chemogenetic activation of excitatory forebrain neurons during postnatal window programs increased anxiety- and despair-like behavior in adulthood (Pati et al., 2020).

It has been hypothesized that early stress may shift the balance toward enhanced excitatory G_q_-coupled signaling accompanied by a decline in inhibitory G_i_-coupled signaling in forebrain neurocircuits, which could contribute to the programming of perturbed anxiety- and despair-like behaviors (Sumner et al., 2008; Lambe et al., 2011; Tiwari et al., 2021). Chemogenetic studies indicate that enhanced G_q_ signaling in forebrain excitatory neurons during postnatal life programs long-lasting increases in anxiety- and despair-like behavior along with disrupted sensorimotor gating (Pati et al., 2020). Several preclinical studies suggest that a loss or reduction in signaling via the G_i_-coupled 5-HT_1A_ receptor during the postnatal temporal window enhances anxiodepressive behaviors in adulthood (Ramboz et al., 1998; Gross et al., 2000, 2002; Vinkers et al., 2010; Richardson-Jones et al., 2010, 2011). However, a recent study indicates that enhanced G_i_ signaling driven chemogenetically in prefrontal cortical neurons during postnatal life results in enhanced adult anxiety- and despair-like behavior, phenocopying the effects of early stress (Teissier et al., 2020). Clinical evidence based on studies of 5-HT_1A_ receptor binding suggests that G_i_-coupled receptors may be associated with resilience to anxiety (Savitz et al., 2009; Armbruster et al., 2011; Albert et al., 2019). Collectively, these reports provide impetus for experiments to test whether perturbation of G_i_ signaling in forebrain excitatory neurons during early developmental windows can alter the programming of mood-related behaviors.

Here, we directly addressed the influence of increased G_i_-mediated signaling in forebrain excitatory neurons in postnatal and juvenile life in the shaping of anxiety- and despair-like behavior, as well as sensorimotor gating responses, in adulthood. We used the G_i_-coupled inhibitory (hM4Di) designer receptors exclusively activated by designer drugs (DREADD), which were expressed in CaMKIIα-positive forebrain excitatory neurons via a bigenic mouse line (CaMKIIα-tTA::TRE hM4Di; Alexander et al., 2009) and used the DREADD ligand clozapine-*N*-oxide (CNO; 5 mg/kg; Roth, 2016) to activate G_i_ signaling during the postnatal window [postnatal day 2 (P2) to P14] and juvenile window (P28 to P40) followed by behavioral analysis in adulthood. We show that hM4Di-DREADD-mediated inhibition of CaMKIIα-positive forebrain excitatory neurons in either the postnatal or juvenile temporal windows does not influence anxiety- and despair-like behavior or sensorimotor gating in adulthood.

## Materials and Methods

### Animals

Bigenic CaMKIIa-tTA::TRE-hM4Di mice were used for all experiments. The CaMKIIa-tTA transgenic mouse line (Mayford et al., 1996) was a gift from Christopher Pittenger (Department of Psychiatry, Yale School of Medicine, New Haven, CT). The TRE-hM4Di mouse line was purchased from The Jackson Laboratory [B6.Cg-Tg(tetO-CHRM4*)2Blr/J; catalog #024114, The Jackson Laboratory]. Bigenic animals were generated for the experiments by mating CaMKIIa-tTA::TRE-hM4Di males to CaMKIIa-tTA::TRE-hM4Di females. The genotypes were confirmed by PCR-based analysis. All dams were individually housed in separate cages, and litter size was restricted to six to eight pups per litter. All animals were bred and maintained in the Tata Institute of Fundamental Research (TIFR; Mumbai, India) animal house facility on a 12 h light/dark cycle from 7:00 A.M. to 7 pm, with *ad libitum* access to food and water. Experimental procedures were conducted as per the guidelines of the Committee for the Purpose of Control and Supervision of Experiments on Animals, Government of India, and were approved by the TIFR animal ethics committee. Care was taken across all experiments to minimize any pain or suffering and to restrict the number of animals used.

### Drug treatment paradigms

For postnatal drug treatments, bigenic CaMKIIα-tTA::TRE-hM4Di mouse pups were orally administered either CNO (catalog #4963, Tocris Bioscience; 5 mg/kg in 5% sucrose solution containing 1% DMSO) or vehicle (5% sucrose solution containing 1% DMSO) for 13 d, commencing from P2 to P14. Postweaning (P24 to P27), animals were group housed for 3 months before assessment on behavioral assays. For juvenile drug treatments, bigenic CaMKIIα-tTA::TRE-hM4Di mouse pups were weaned between P24 and P27, group housed, and randomly assigned to either the vehicle or CNO treatment groups. Juvenile bigenic CaMKIIα-tTA::TRE-hM4Di mice received either CNO (5 mg/kg in 5% sucrose solution containing 1% DMSO) or vehicle (5% sucrose solution containing 1% DMSO) for 13 d from P28 to P40. All animals were left undisturbed from P41 for 2 months before subjecting them to behavioral testing. To assess whether postnatal CNO (PNCNO) or juvenile CNO (JCNO) treatment influenced the weight of pups, we conducted an extensive weight profiling across the duration of the PNCNO and JCNO treatment paradigms. We chose the treatment time windows to reflect a postnatal window in which early stress paradigms are performed, and a juvenile window postweaning.

### Western blotting

To assess HA-tagged hM4Di-DREADD expression in the hippocampus and cortex of CaMKIIα-tTA::TRE-hM4Di bigenic mice at P7 and P35, we conducted Western blotting analysis for the HA antigen. To determine the influence of CNO-mediated activation of the hM4Di-DREADD on neuronal activity marker expression (c-Fos), we administered a single dose of CNO (5 mg/kg) or vehicle to CaMKIIα-tTA::TRE-hM4Di bigenic mouse pups at P7 and to the juvenile cohort at P35, and killed them 30 min postadministration. Hippocampi and cortex tissue were dissected and homogenized in radioimmunoprecipitation assay buffer (50 nm Tris-Cl, pH 8.0, 5 mm EDTA, 1% NP-40, 150 mm NaCl) using a dounce homogenizer. Protein concentration was estimated with the QuantiPro BCA Assay Kit (Sigma-Aldrich), and lysates were resolved on a 10% SDS polyacrylamide gel before transfer onto polyvinylidene fluoride membranes. Blots were subjected to blocking in 5% milk in TBST and incubated overnight with the following respective primary antibodies: rabbit anti-HA (1:1500; catalog #H6908, Sigma-Aldrich); rabbit anti-c-Fos (1:1000; catalog #2250, Cell Signaling Technology); and rabbit anti-β-actin (1:10,000; catalog #AC026, ABclonal Technology). Blots were exposed to HRP-conjugated goat anti-rabbit secondary antibody (1:6000; catalog #AS014, ABclonal Technology) for 1 h with signal visualized on an Imager 600 (GE Healthcare) using a Western blotting detection kit (WesternBright ECL, Advansta). Densitometric quantitative analysis was performed using ImageJ software.

### Immunofluorescence analysis

Coronal brain sections (40 μm) were generated on a vibratome (Leica) from adult CaMKIIα-tTA::TRE-hM4Di bigenic mice killed by transcardial perfusion with 4% paraformaldehyde. Sections were permeabilized and blocked in PBS with 0.3% Triton X-100 (PBSTx) containing 10% horse serum (catalog #26–050-088, Thermo Fisher Scientific) for 2 h at room temperature. The sections were then incubated with primary antibody for double-labeled immunofluorescence experiments to examine the colocalization of the HA-tagged hM4Di-DREADD with markers for excitatory and inhibitory neurons, and glial cells in the hippocampus and neocortex. The following antibody cocktails were used: rat anti-HA (1:200; catalog #10145700, Roche Diagnostics) with rabbit anti-CaMKIIα (1:200; catalog #sc-12 886-R, Santa Cruz Biotechnology), mouse anti-parvalbumin (PV; 1:500; catalog #P3088, Sigma-Aldrich), or rabbit anti-glial fibrillary acidic protein (GFAP; 1:500; catalog #AB5804, Chemicon) for 4 d at 4°C. This was followed by the washing of sections with 0.3% PBSTx three times for 15 min each. The sections were then incubated with the following cocktails of secondary antibodies: goat anti-rat IgG conjugated to Alexa Fluor 488 (1:500; catalog #A-21212, Thermo Fisher Scientific) and goat anti-rabbit IgG conjugated to Alexa Fluor 568 (1:500; catalog #A-11011, Thermo Fisher Scientific), or goat anti-rat IgG conjugated to Alexa Fluor 488 (1:500; catalog #A-21212, Thermo Fisher Scientific) and donkey anti-mouse IgG conjugated to Alexa Fluor 555 (1:500; catalog #A-31570, Thermo Fisher Scientific) for 2 h at room temperature. After sequential washing with 0.3% PBSTx, sections were mounted on slides using Vectashield Antifade Mounting Medium with DAPI (catalog #H-1200, Vector Laboratories) and images were visualized on a confocal microscope (model FV1200, Olympus).

### Behavioral assays

Reflex behaviors for neonates were analyzed on CaMKIIα-tTA::TRE-hM4Di bigenic mouse pups commencing on P9 till P12, with air righting, surface righting, and negative geotaxis determined.

#### Air righting

Animals were allowed to fall 10 times from a height of 25 cm, facing upside down. The number of correct landings, as observed by falling on all four paws, was determined.

#### Negative geotaxis

Animals were placed on an inclined plank (30°), facing downward. The length of time taken by the animal to turn (180°) and face upward was noted.

#### Surface righting

The time for the pup to attain a standing position with all four paws was noted when placed upside down in the home cage.

In adulthood, CaMKIIα-tTA::TRE-hM4Di bigenic mice were subjected to behavioral assays to assess anxiety-like behavior [open field test (OFT); elevated plus maze (EPM) test; light-dark avoidance test (LD box)] despair-like behavior [tail suspension test (TST) and forced swim test (FST)], and sensorimotor gating behavior, which was assessed via the prepulse inhibition (PPI) test. All anxiety-like behavioral assays were recorded and tracked using an overhead camera at 25 frames/s. All despair-like behaviors were recorded using a side-mounted webcam (Logitech). Behavior tracking was performed using the automated platform Ethovision XT 11.

#### Open field test

Mice were released into one corner (chosen at random) of the open field arena (40 × 40 × 40 cm), and were allowed to explore for 10 min. The total distance moved in the arena, the percentage of time spent and the percentage of distance in the center, and the number of entries to the center were determined.

#### Elevated plus maze

Animals were introduced to the elevated plus maze for 10 min and were placed in the center of the plus maze facing the open arms. The elevated plus maze was built such that the two arms both open and closed (30 × 5 cm each) were elevated 50 cm above the ground. The walls of the closed arms were 15 cm high. The total distance moved in the maze, the percentage of time spent, the percentage of distance, and the number of entries in the open and closed arms were determined.

#### Light-dark avoidance test

The LD box was made of two joint chambers: the light chamber (25 × 25 cm) and the dark chamber (15 × 25 cm). The two areas were connected by an opening (10 × 10 cm). Mice were released into the arena facing the light chamber at the cusp of the lit and dark arenas for a duration of 10 min. The number of entries and the percentage of time spent in the light arena were assessed.

#### Tail suspension test

Animals were suspended by their tail for 6 min at a height of 50 cm above the ground, and the total immobility time and number of immobility events were assessed for a duration of 5 min, excluding the first minute of the test.

#### Forced swim test

Animals were allowed to swim for 6 min in a transparent cylinder 50 cm in height with a 14 cm inner diameter that was filled with water (25°C) up to a height of 30 cm. The total immobility time and the number of immobility events were determined for a duration of 5 min excluding the first minute of the test.

#### Prepulse inhibition test

Animals were assayed for perturbation of sensorimotor gating on the prepulse inhibition test performed using a startle and fear conditioning apparatus (Panlab). CaMKIIα-tTA::TRE-hM4Di bigenic mice were allowed to habituate to the restrainer and testing apparatus for 15 min daily across 4 d, followed by a 15 min habituation per day for 4 d with an exposure to 65 dB background white noise. On the test day, following exposure of the animals to 65 dB background white noise for 5 min, 10 tone pulses (120 dB, 1 s) were presented to the mice to measure basal startle response (first block). The mice were then randomly presented with either only tone (120 dB, 1 s, 10×) or a 100 ms prepulse that was +4 dB (69 dB), +8 dB (73 dB), or +16 dB (81 dB) higher than the background noise, five times each, which coterminated with a 1 s, 120 dB tone. The percentage of PPI was calculated using the following formula: percentage PPI = 100 × (average startle response with only tone − average startle response with the prepulse)/average startle response with only tone.

### Statistical analysis

The Kolmogorov–Smirnov test was used to confirm the normality of distribution. All experiments that had two treatment groups were subjected to a two-tailed, unpaired Student’s *t* test using GraphPad Prism (GraphPad Software). A Welch correction was applied when a significant difference in the variance between groups was observed. Experiments with four treatment groups were subjected to a two-way ANOVA with PNCNO and sex as the two variables. The Tukey’s *post hoc* comparison tests were performed only when a significant two-way ANOVA interaction of PNCNO × sex interaction was observed. Data are expressed as the mean ± SEM, and statistical significance was set at *p *<* *0.05.

## Results

### Selective expression of hM4Di-DREADD in CaMKIIα-positive forebrain excitatory neurons in CaMKIIα-tTA::TRE-hM4Di bigenic mice

**Figure 1.**
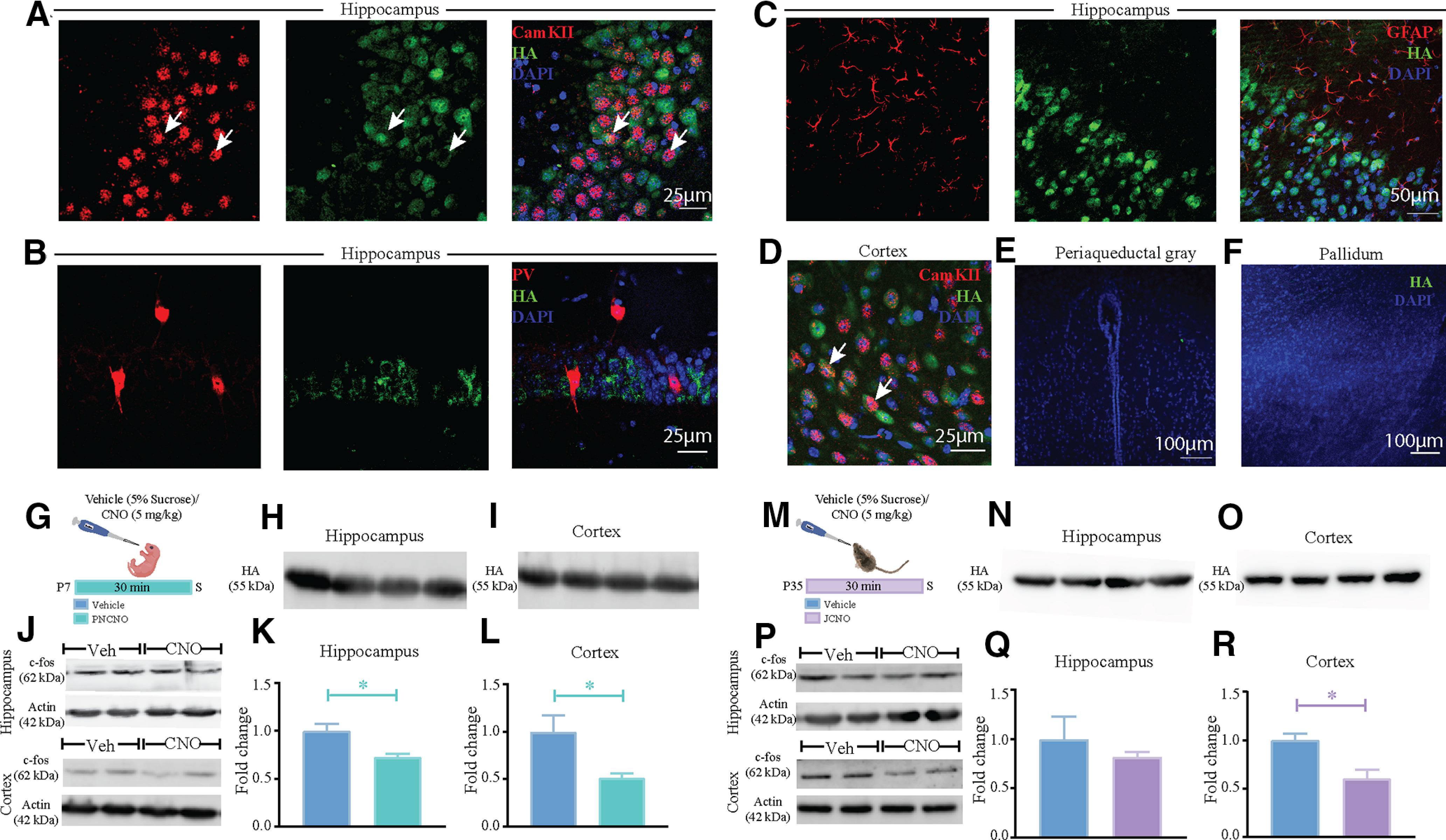
Selective expression of hM4Di-DREADD in CaMKIIα-positive forebrain excitatory neurons in CaMKIIα-tTA::TRE-hM4Di bigenic mice. ***A***, Shown are representative confocal images indicating expression of the HA-tagged hM4Di-DREADD in the hippocampus as identified by HA/CaMKIIα double immunofluorescence. ***B***, ***C***, HA-tagged hM4Di-DREADD expression was not observed in either PV-positive inhibitory interneurons (***B***) or GFAP-positive astrocytes (***C***). ***D***, HA-tagged hM4Di-DREADD in the cortex was also observed in the CaMKIIα-positive neurons as identified with HA/CaMKIIα double immunofluorescence. ***E***, ***F***, Immunofluorescence experiments indicate the absence of expression of HA-tagged hM4Di-DREADD in subcortical brain regions, namely the periaqueductal gray (***E***) and pallidum (***F***). ***G***, Shown is a schematic of the experimental paradigm for harvesting cortex and hippocampus at P7 for Western blotting analysis. ***H***, ***I***, HA expression was clearly noted in the cortex (***H***) as well as the hippocampus (***I***) in Western blots from CaMKIIα-tTA::TRE-hM4Di bigenic pups (P7). ***J***, Shown are representative Western blots for c-Fos along with their respective actin loading controls at P7 half an hour after vehicle (Veh) or CNO treatment for cortex (top panel) and hippocampus (bottom panel). ***K***, ***L***, Quantitative densitometry indicated a significant reduction in c-Fos protein levels in the cortex (***K***) as well as the hippocampus (***L***) of PNCNO-treated bigenic pups at P7 compared with their vehicle-treated controls. ***M***, Shown is a schematic of the experimental paradigm for harvesting cortex and hippocampus at P35 in the juvenile window for Western blotting analysis. ***N***, ***O***, HA expression was noted in the cortex (***N***) as well as the hippocampus (***O***) of CaMKIIα-tTA::TRE-hM4Di bigenic juvenile mice (P35). ***P***, Shown are representative Western blots for c-Fos along with their respective actin loading controls at P35 half an hour after vehicle (Veh) or CNO treatment for cortex (top panel) and hippocampus (bottom panel). ***Q***, ***R***, Quantitative densitometry indicated a significant reduction in c-Fos protein levels in the cortex (***Q***) but not in the hippocampus (***R***) of JCNO-treated bigenic mice at P35 compared with their vehicle-treated controls. All immunofluorescence experiments and Western blotting experiments were performed on *n* = 3–5/group. Results are expressed as the mean ± SEM. *p <* *0.05, compared with vehicle-treated controls using the two-tailed, unpaired Student’s *t* test.

To address the behavioral consequences of hM4Di-DREADD-mediated inhibition of forebrain excitatory neurons in postnatal and juvenile windows of life, CaMKIIα-tTA::TRE-hM4Di bigenic mice were generated. The expression of the HA-tagged hM4Di-DREADD was characterized in the hippocampus and cortex, wherein the CaMKIIα-tTA driver would result in expression in forebrain excitatory neurons (Mayford et al., 1996; Wang et al., 2013), as well as within brain regions such as the periaqueductal gray (PAG) and pallidum, which are known to lack an expression of CaMKIIα-positive neurons. The presence of the HA-tagged hM4Di-DREADD was confirmed in CaMKIIα-positive neurons in both the hippocampus (Fig. 1*A*) and the cortex (Fig. 1*D*) of adult CaMKIIα-tTA::TRE-hM4Di bigenic mice. The HA-tagged hM4Di-DREADD was not present on the inhibitory PV-positive neurons (Fig. 1*B*), as well as in GFAP-immunopositive astrocytes (Fig. 1*C*) in the hippocampus. We also confirmed the absence of the HA-tagged hM4Di-DREADD in select brain regions that lack CaMKIIα-positive neurons, namely the PAG (Fig. 1*E*) and pallidum (Fig. 1*F*).

We next examined the presence of the HA-tagged hM4Di-DREADD in the hippocampus and cortex using Western blotting analysis. CaMKIIα-tTA::TRE-hM4Di bigenic mice at P7 (Fig. 1*G–I*) as well as in juveniles at P35 (Fig. 1*M–O*) exhibited robust expression of the HA-tagged hM4Di-DREADD. We then performed Western blotting analysis for the neuronal activity marker c-Fos to examine whether hM4Di-DREADD evoked a reduction in neuronal activity in the hippocampus and cortex, half an hour post-CNO or vehicle treatment to postnatal pups at P7 and juvenile animals at P35. Western blotting analysis, followed by quantitative densitometry for c-Fos protein levels revealed a significant reduction in the hippocampus (Fig. 1*K*) and cortex (Fig. 1*L*) of PNCNO-treated CaMKIIα-tTA::TRE-hM4Di bigenic mouse pups. For the JCNO-treated CaMKIIα-tTA::TRE-hM4Di bigenic mice at P35, we did not observe a change in the c-Fos protein levels in the hippocampus (Fig. 1*Q*), but did note a significant decline in c-Fos protein levels in the cortex (Fig. 1*R*) of the JCNO-treated cohort. Collectively, these results indicate that the expression of HA-tagged hM4Di-DREADD is restricted to forebrain CaMKIIα-positive neurons, and that treatment with the DREADD ligand CNO in the early postnatal or juvenile window evokes a decline in activity of within the forebrain regions of the hippocampus and cortex, as indicated by a reduction in protein levels of the neuronal activity marker c-Fos.

### Chronic hM4Di-DREADD-mediated inhibition of CaMKIIα-positive forebrain excitatory neurons during the early postnatal window does not influence anxiety-like behavior in adulthood in male or female mice

**Figure 2.**
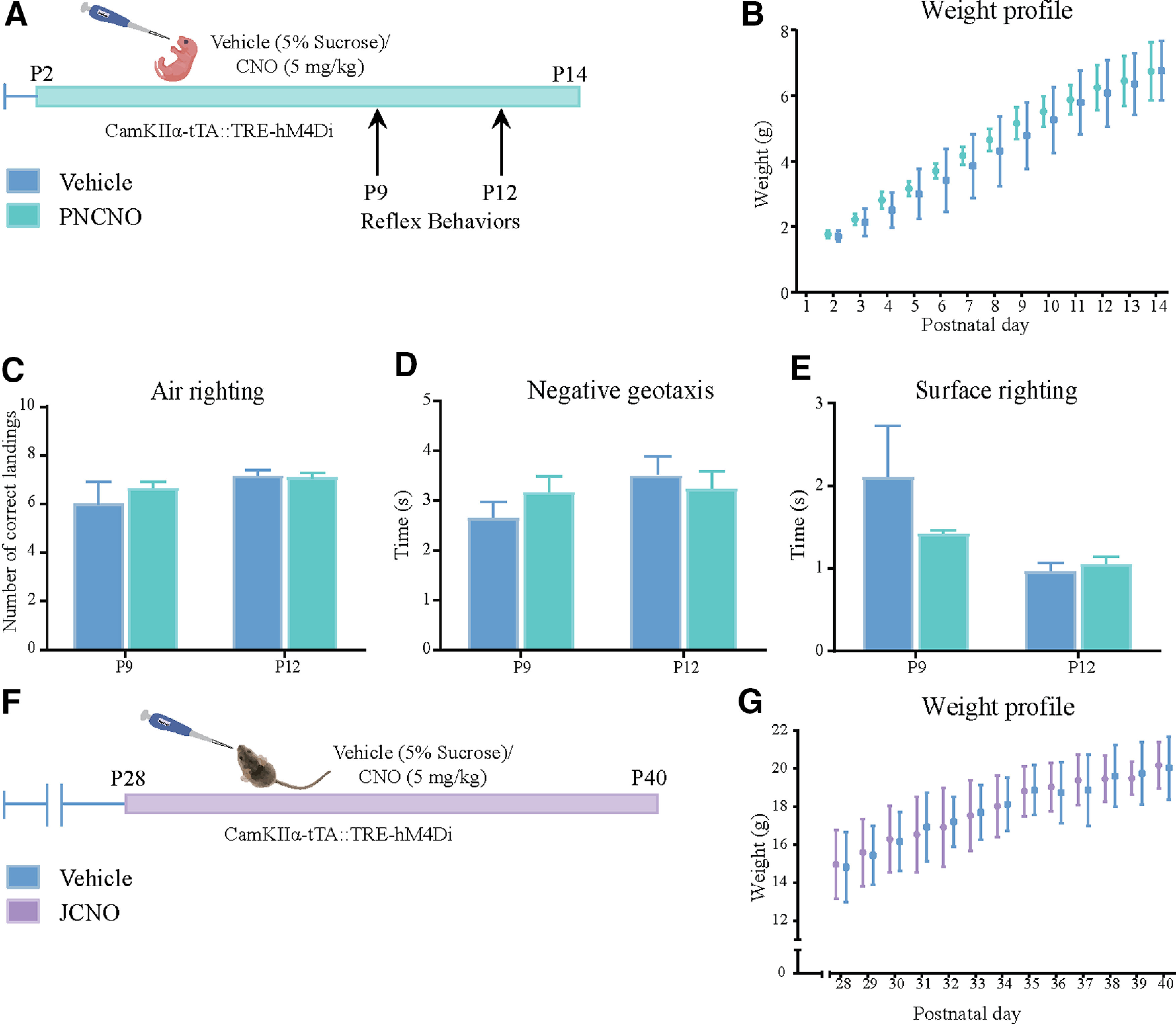
Influence of chronic hM4Di-DREADD-mediated inhibition of CaMKIIα-positive forebrain neurons in the early postnatal or juvenile windows on weight and reflex development. ***A***, Shown is a schematic for the experimental paradigm for vehicle (5% sucrose) or CNO (5 mg/kg) administration in the early postnatal window (P2 to P14) in CaMKIIα-tTA::TRE-hM4Di bigenic pups. Pups were assessed for weight gain across the postnatal developmental window and for reflex behaviors on postnatal days 9 and 12. ***B***, No significant change was observed in the weight profile of CNO-administered pups compared with their vehicle-treated age-matched controls across the duration of CNO treatment from P2 to P14 (*n* = 6). ***C–E***, Reflex behaviors were not altered in PNCNO-treated CaMKIIα-tTA::TRE-hM4Di bigenic pups compared with vehicle-treated controls at P9 or P12 as assessed by determining the number of correct landings for air righting (***C***), and the time taken for reorientation in both negative geotaxis (***D***) and surface righting (***E***) assays. ***F***, Shown is a schematic for the experimental paradigm for vehicle (5% sucrose) or CNO (5 mg/kg) administration in the early juvenile window (P28 to P40) to CaMKIIα-tTA::TRE-hM4Di bigenic male mice. ***G***, No significant change was noted in the weight profile of animals fed with CNO (5 mg/kg) once daily from P28 to P40 compared with their vehicle-treated controls across the duration of drug treatment (*n* = 5–6/group). Results are expressed as the mean ± SEM, and groups are compared using the two-tailed, unpaired Student’s *t* test.

We set out to examine the behavioral influence of chronic CNO-mediated hM4Di-DREADD inhibition of CaMKIIα-positive forebrain excitatory neurons during the early postnatal or juvenile window by orally administering the DREADD ligand CNO (5 mg/kg) or vehicle to CaMKIIα-tTA::TRE-hM4Di bigenic male and female mice once daily from P2 to P14 (Fig. 2*A*; PNCNO) or from P28 to P40 (Fig. 2*F*; JCNO). PNCNO or JCNO treatments did not alter body weight, which was measured across the period of treatment (Fig. 2*B*,*G*). PNCNO treatment did not alter the normal ontogeny of reflex behaviors, namely air righting (Fig. 2*C*), negative geotaxis (Fig. 2*D*), and surface righting (Fig. 2*E*), in PNCNO-treated CaMKIIα-tTA::TRE-hM4Di bigenic mouse pups compared with their vehicle-treated controls.

**Figure 3.**
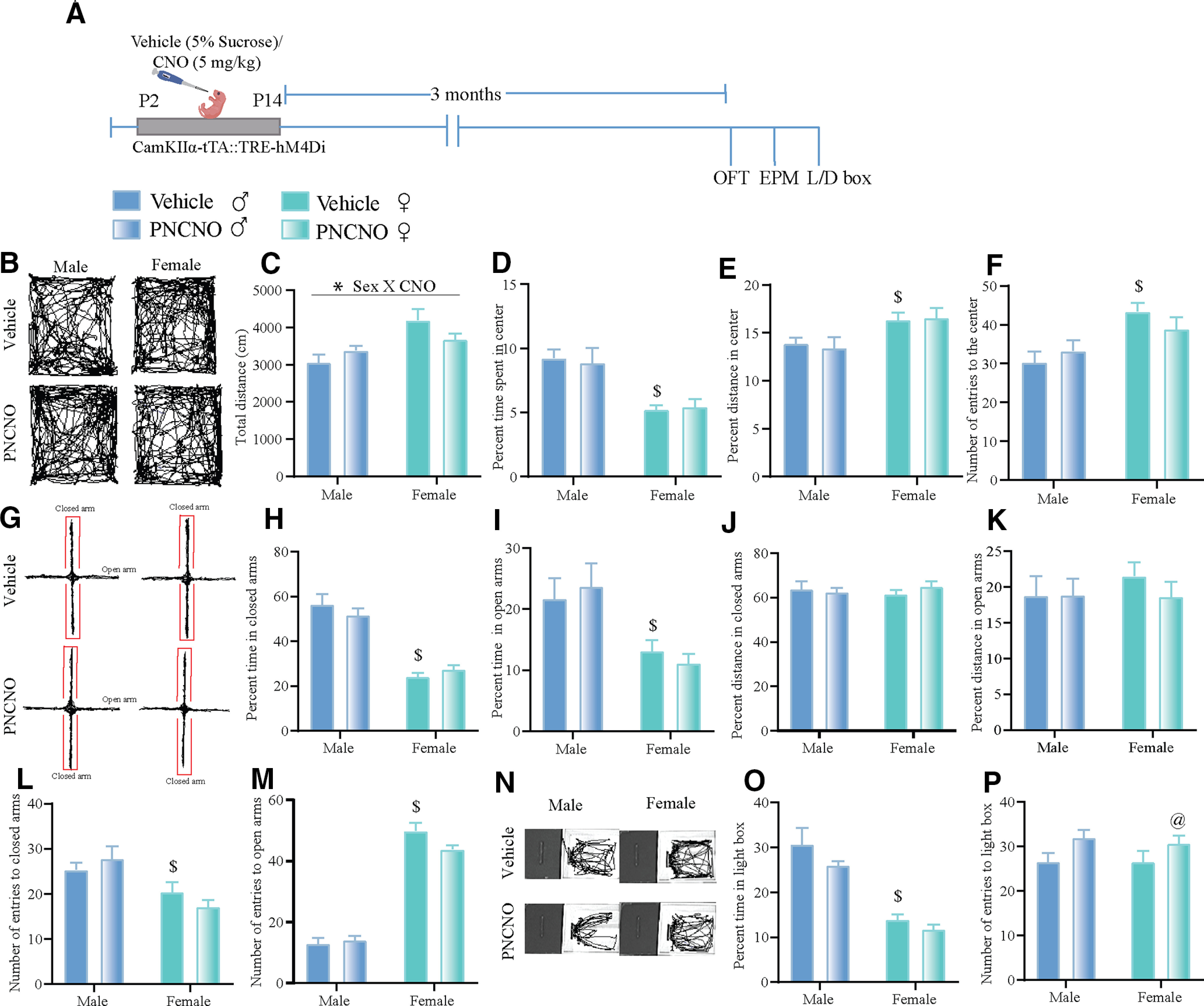
Chronic hM4Di-DREADD-mediated inhibition of CaMKIIα-positive forebrain excitatory neurons during the early postnatal window does not influence anxiety-like behavior in adulthood in CaMKIIα-tTA::TRE-hM4Di bigenic mice. ***A***, Shown is a schematic for the experimental paradigm for vehicle (5% sucrose) or CNO (5 mg/kg) administration in the early postnatal window (P2 to P14) to CaMKIIα-tTA::TRE-hM4Di bigenic pups, which were then assessed for anxiety-like behaviors 3 months postcessation of CNO treatment in adulthood. ***B***, Shown are representative tracks for vehicle-treated (top panels) and PNCNO-treated (bottom panels) CaMKIIα-tTA::TRE-hM4Di bigenic male and female mice in the open field arena. ***C***, Two-way ANOVA indicated a PNCNO × sex interaction for total distance moved in the OFT arena, with *post hoc* Tukey’s analysis revealing a significant difference between the vehicle-treated female and male CaMKIIα-tTA::TRE-hM4Di bigenic mice. ***D–F***, We noted significant main effects of sex for the percentage of time spent in the center (***D***), the percentage of the distance traveled in the center (***E***), and the total number of entries to the center of the open field arena (***F***). *n* = 10 (males) and *n* = 10 (females) for vehicle-treated CaMKIIα-tTA::TRE-hM4Di bigenic mice; *n* = 12 (males), *n* = 12 (females) for PNCNO-treated CaMKIIα-tTA::TRE-hM4Di bigenic mice. ***G***, Shown are representative tracks for vehicle-treated (top panels) and PNCNO-treated (bottom panels) CaMKIIα-tTA::TRE-hM4Di bigenic male and female mice in the elevated plus maze. ***H–M***, We noted a significant main effect of sex for the percentage of time in the closed (***H***) and open (***I***) arms of the plus maze, and for the number of entries to the closed (***L***) and open (***M***) arms, but not for the percentage of distance traveled in closed (***J***) or open (***K***) arms. *n* = 14 (males) and *n* = 10 (females) for vehicle-treated CaMKIIα-tTA::TRE-hM4Di bigenic mice; *n* = 12 (males) and *n* = 12 (females) for PNCNO-treated CaMKIIα-tTA::TRE-hM4Di bigenic mice. ***N***, Shown are representative tracks for vehicle-treated (top panels) and PNCNO-treated (bottom panels) CaMKIIα-tTA::TRE-hM4Di bigenic mice in the LD box. No significant interaction of PNCNO × sex was noted in the LD box. ***O***, ***P***, However, we did observe a significant main effect of sex in the total time spent in the light box (***O***), and a significant main effect of PNCNO treatment on the total number of entries into the light chamber of the LD box (***P***). *n* = 14 (males) and *n* = 10 (females) for vehicle-treated CaMKIIα-tTA::TRE-hM4Di bigenic mice; *n* = 12 (males) and *n* = 12 (females) PNCNO-treated CaMKIIα-tTA::TRE-hM4Di bigenic mice. Results are expressed as the mean ± SEM, and groups are compared using two-way ANOVA, followed by the Tukey’s *post hoc* comparison test when a significant PNCNO × sex interaction was noted (*p < 0.05). The main effects of sex are indicated as ^$^*p* < 0.05; the main effects of CNO treatment are indicated as ^@^*p* < 0.05.

We next addressed whether a history of hM4Di-DREADD-mediated inhibition of CaMKIIα-positive forebrain excitatory neurons during the early postnatal window alters anxiety-like behavior in adulthood. We subjected CaMKIIα-tTA::TRE-hM4Di bigenic adult male and female mice with a history of PNCNO treatment to a battery of behavioral tests to assess anxiety-like behavior, namely the OFT, EPM test, and LD box test. We do not observe any significant alterations in multiple behavioral measures in the OFT between the vehicle- and PNCNO-treated cohorts in both male and female (Fig. 3*B–F*) CaMKIIα-tTA::TRE-hM4Di bigenic mice. We noted a significant PNCNO × sex interaction (*F*_(1,40)_ = 4.318, *p *=* *0.044) in the total distance traveled (Fig. 3*C*) in the OFT arena, with Tukey’s *post hoc* comparisons revealing a significant difference between vehicle-treated male and female CaMKIIα-tTA::TRE-hM4Di bigenic mice. No significant interactions between PNCNO and sex were noted for the other measures assessed in the OFT. We did note a significant main effect of sex for percentage of time spent in the center (*F*_(1,40)_ = 21.77, *p *<* *0.0001; Fig. 3*D*), percentage of the distance traveled in the center (*F*_(1,40)_ = 8.727, *p *=* *0.0052; Fig. 3*E*), and the number of entries to the center (*F*_(1,40)_ = 11.07, *p *=* *0.002; Fig. 3*F*). We noted no significant effect of PNCNO for any of the behavioral measures assessed in the OFT.

We did not observe any significant PNCNO × sex interaction for the multiple behavioral measures assessed in the EPM test (Fig. 3*G–M*). We observed a significant main effect of sex for the percentage of time spent in closed arms (*F*_(1,45)_ = 76.53, *p *<* *0.0001; Fig. 3*H*) and the percentage of time spent in open arms (*F*_(1,45)_ = 12.98, *p *=* *0.0008; Fig. 3*I*), as well as for the number of entries to closed arms (*F*_(1,45)_ = 14.38, *p *=* *0.0004; Fig. 3*L*) and open arms (*F*_(1,45)_ = 290.7, *p *< 0.0001; Fig. 3*M*), but not for the measures of percentage of distance traveled in closed (Fig. 3*J*) and open (Fig. 3*K*) arms. We noted no significant effect of PNCNO for any of the behavioral measures assessed in the EPM test. We next assessed the behavior of vehicle- and PNCNO-treated CaMKIIα-tTA::TRE-hM4Di bigenic adult male and female mice on the LD box. We noted no significant PNCNO × sex interactions for either the percentage of time or the number entries in the light box. We noted a significant main effect of sex for percentage of time spent in the light box (*F*_(1,42)_ = 40.27, *p *<* *0.0001; Fig. 3*O*), and a significant main effect of PNCNO for the number of entries to the light box (*F*_(1,42)_ = 5.227, *p *=* *0.027; Fig. 3*P*). Together, these findings indicate that PNCNO-mediated, chronic hM4Di-DREADD inhibition of CaMKIIα-positive forebrain excitatory neurons during the early postnatal window does not appear to significantly influence anxiety-like behavior in adulthood on the OFT, EPM test, and LD test in both male and female CaMKIIα-tTA::TRE-hM4Di bigenic mice. However, we do note robust main effects of sex on multiple measures across distinct anxiety-related behavioral tasks.

### Chronic hM4Di-DREADD-mediated inhibition of CaMKIIα-positive forebrain excitatory neurons during the early postnatal window does not influence adult despair-like behavior in male and female mice or sensorimotor gating responses in male mice

**Figure 4.**
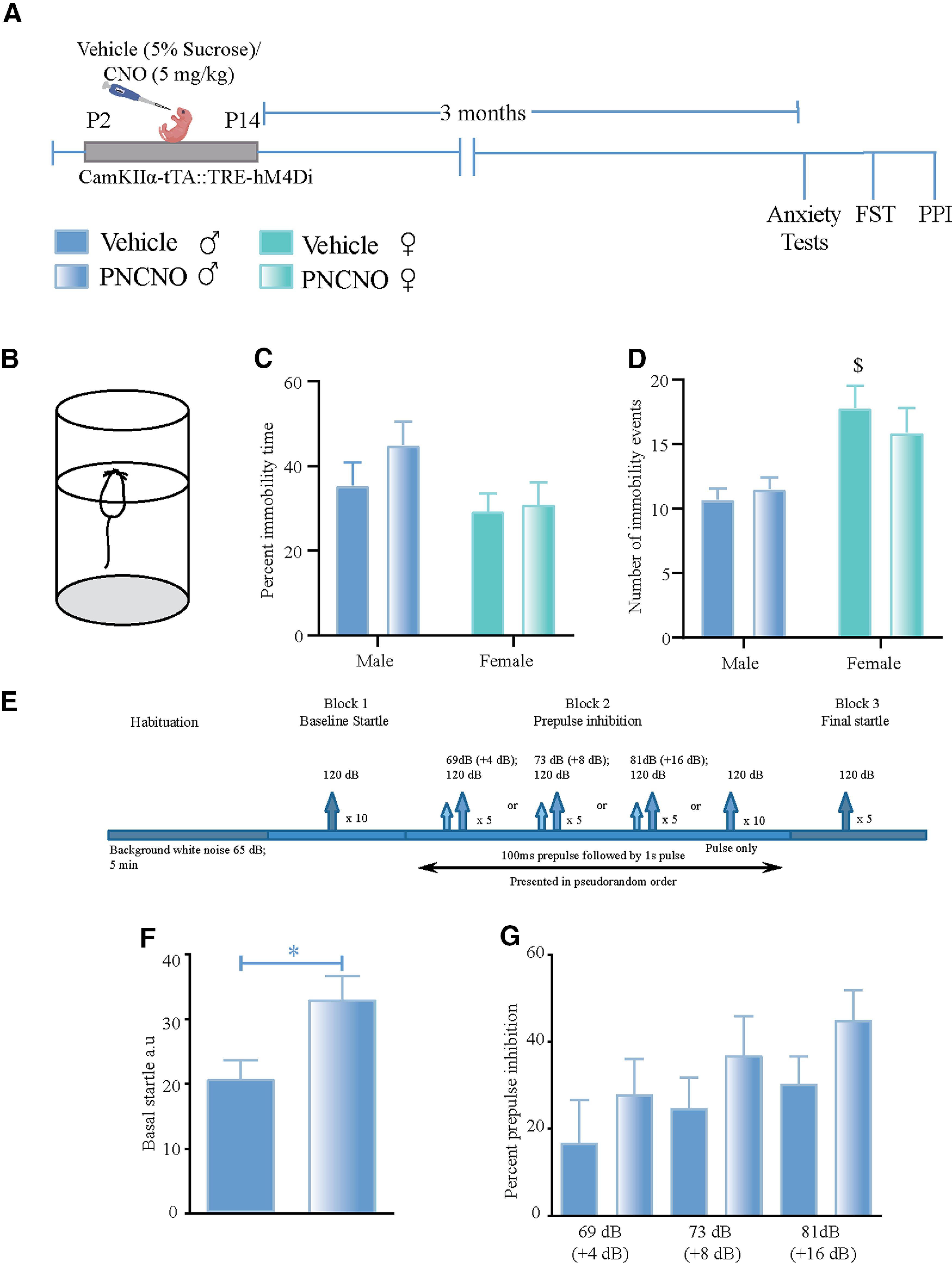
Chronic chemogenetic inhibition of CaMKIIα-positive forebrain excitatory neurons during the early postnatal window does not influence despair-like behavior or sensorimotor gating responses in adult mice. ***A***, Shown is a schematic for the experimental paradigm for vehicle (5% sucrose) or CNO (5 mg/kg) administration in the early postnatal window (P2 to P14) to CaMKIIα-tTA::TRE-hM4Di bigenic pups, which were then assessed for despair-like behavior in adulthood using the FST and sensorimotor gating responses (PPI). ***B***, Shown is a schematic for the FST tank. ***C***, ***D***, We observed no significant PNCNO × sex interactions for either the percentage of immobility time (***C***) or the total number of immobility events [***D***; *n* = 14 (males), *n* = 10 (females) for vehicle-treated CaMKIIα-tTA::TRE-hM4Di bigenic mice; *n* = 12 (males), *n* = 10 (females) for PNCNO-treated CaMKIIα-tTA::TRE-hM4Di bigenic mice]. ***D***, We did note a significant main effect of sex for the number of immobility events. ***E***, Shown is a schematic for the protocol used for PPI to assess sensorimotor gating responses in adult male mice. PPI testing was conducted as described in Materials and Methods with basal startle determined following habituation, and PPI determined for +4 dB (69 dB), +8 dB (73 dB), and +16 dB (81 dB) above background noise (65 dB), followed by exposure to 120 dB for final startle. ***F***, PNCNO-treated adult CaMKIIα-tTA::TRE-hM4Di bigenic male mice show a significant increase in basal startle response compared with vehicle-treated controls. ***G***, No significant differences were noted in sensorimotor gating between vehicle- and PNCNO-treated CaMKIIα-tTA::TRE-hM4Di bigenic male mice (*n* = 10/group). Results were subjected to two-way ANOVA, followed by the Tukey’s *post hoc* comparisons test for experiments with four treatment groups (main effects of sex are indicated as ^$^*p* < 0.05), and by the two-tailed, unpaired Student’s *t* test for experiments with two treatment groups. Results are expressed as the mean ± SEM. *p < 0.05 compared with the vehicle-treated controls.

We next addressed whether a history of hM4Di-DREADD-mediated inhibition of CaMKIIα-positive forebrain excitatory neurons during the early postnatal window alters despair-like behavior in adulthood on the FST (Fig. 4*A–D*). PNCNO treatment did not alter either the percentage of immobility time or the number of immobility events, indicating no change in despair-like behavior in CaMKIIα-tTA::TRE-hM4Di bigenic adult male and female mice. We did not observe any significant PNCNO × sex interaction for the percentage of immobility time (Fig. 4*C*) or the number of immobility events (Fig. 4*D*). We did note a significant effect of sex on the number of immobility events (*F*_(1,42)_ = 18.45, *p *=* *0.0001). We noted no significant main effect of PNCNO on either of the measures assessed in the FST. We also performed TST to assess despair-like behavior in adult vehicle- and PNCNO-treated CaMKIIα-tTA::TRE-hM4Di bigenic male mice and observed no change in despair-like behavior (percentage of immobility time: vehicle-treated CaMKIIα-tTA::TRE-hM4Di bigenic adult male mice = 60.54 ± 4.5, *n* = 8; PNCNO-treated CaMKIIα-tTA::TRE-hM4Di bigenic adult male mice = 62.56 ± 2.63, *n* = 9; results are expressed as the mean ± SEM).

Disruption of excitation/inhibition balance in the neocortex has been linked to altered schizoaffective behavior in adulthood (Yizhar et al., 2011; Fenton, 2015; Marín, 2016; Anticevic and Lisman, 2017; Pati et al., 2020). We sought to address whether chronic CNO-mediated hM4Di inhibition of CaMKIIα-positive forebrain excitatory neurons in the early postnatal window resulted in any change in sensorimotor gating behavior in adulthood. To measure changes in sensorimotor gating, we subjected PNCNO-treated CaMKIIα-tTA::TRE-hM4Di bigenic male mice and their respective vehicle-treated control groups to the PPI paradigm (Fig. 4*E*). We noted no significant difference in the percentage of prepulse inhibition, at all prepulse tones tested above the background noise, between the PNCNO-treated CaMKIIα-tTA::TRE-hM4Di bigenic male mice and their respective vehicle-treated controls (Fig. 4*G*). However, we did observe a significant increase in basal startle response in the CaMKIIα-tTA::TRE-hM4Di bigenic male mice with a history of PNCNO treatment when compared with their vehicle-treated controls (Fig. 4*F*). Collectively, these results indicate that chronic hM4Di-DREADD-mediated chemogenetic inhibition of CaMKIIα-positive forebrain excitatory neurons during the early postnatal does not alter despair-like behavior on the FST or TST in adulthood, and does not result in any significant change in sensorimotor gating, but may evoke perturbed baseline startle responses in the PNCNO-treated group.

### Chronic hM4Di-DREADD-mediated inhibition of CaMKIIα-positive forebrain excitatory neurons during the juvenile window does not influence anxiety, despair, or sensorimotor gating behavior in adulthood

**Figure 5.**
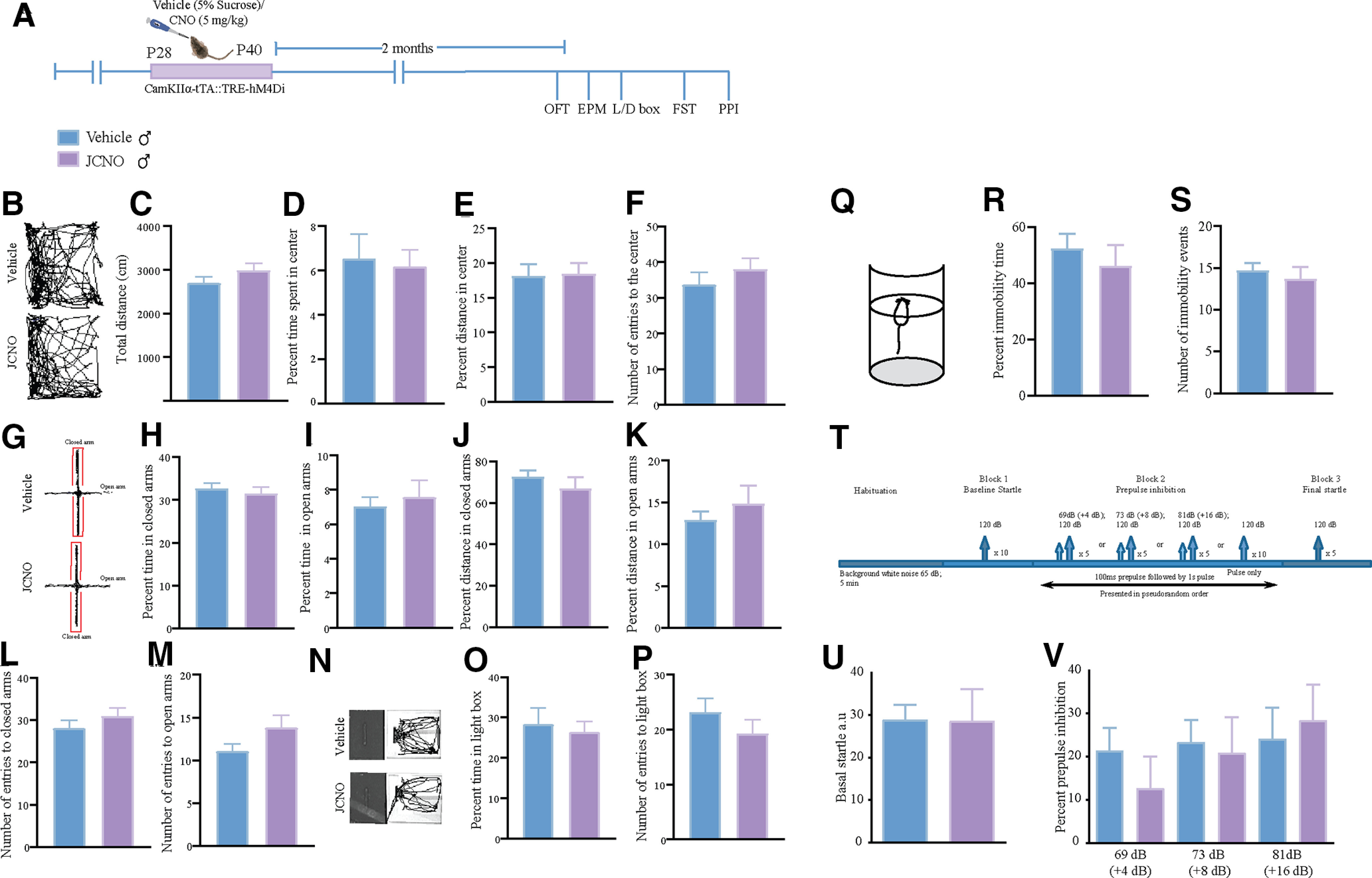
Chronic hM4Di-DREADD-mediated inhibition of CaMKIIα-positive forebrain excitatory neurons during the juvenile window does not influence anxiety, despair, or sensorimotor gating behavior in adulthood in CaMKIIα-tTA::TRE-hM4Di bigenic male mice. ***A***, Shown is a schematic for the experimental paradigm for vehicle (5% sucrose) or CNO (5 mg/kg) administration in the juvenile window (P28 to P40) to CaMKIIα-tTA::TRE-hM4Di bigenic mice, which were then assessed for anxiety-like behaviors 2 months postcessation of CNO treatment in adulthood in the male cohort. ***B***, Shown are representative tracks for vehicle-treated (top panel) and JCNO-treated CaMKIIα-tTA::TRE-hM4Di bigenic male mice (bottom panel) in the open field arena. ***C–F***, No significant difference was noted between vehicle- and JCNO-treated male mice in the total distance traveled in the arena (***C***), the percentage of time spent in the center (***D***), the percentage of distance traveled in the center (***E***), or the total number of entries to the center of the open field arena (***F***; *n* = 18 for vehicle-treated male mice; *n* = 16 for JCNO-treated male mice). ***G***, Shown are representative tracks for vehicle-treated (top panel) and JCNO-treated CaMKIIα-tTA::TRE-hM4Di bigenic male mice (bottom panel) in the elevated plus maze. ***H–M***, No significant difference was observed between vehicle- and JCNO-treated male mice in the percentage of time spent in the closed (***H***) or open (***I***) arms of the EPM, as well as the percentage of distance traveled in closed (***J***) or open (***K***) arms, and for the number of entries to the closed (***L***) or open (***M***) arms (*n* = 18 for vehicle-treated male mice; *n* = 16 for JCNO-treated male mice). ***N***, Shown are representative tracks for vehicle-treated (top panel) and JCNO-treated (bottom panel) CaMKIIα-tTA::TRE-hM4Di bigenic male mice in the LD box. ***O***, ***P***, No significant difference was noted between vehicle- and JCNO-treated male mice in either the total time spent in (***O***) or the total number of entries (***P***) into the light chamber of the LD box (*n* = 18 for vehicle-treated male mice; *n* = 16 for JCNO-treated male mice). ***Q***, Shown is a schematic for the FST tank. ***R***, ***S***, No significant difference was noted between vehicle- and PNCNO-treated male mice for the percentage of immobility time (***R***) or for the total number of immobility events (***S***; *n* = 11 for vehicle-treated male mice; *n* = 11 for JCNO-treated male mice). ***T***, Shown is a schematic for the protocol used for PPI to assess sensorimotor gating responses in adult male mice. PPI testing was conducted as described in Materials and Methods with basal startle determined following habituation, and PPI determined for +4 dB (69 dB), +8 dB (73 dB), and +16 dB (81 dB) above background noise (65 dB), followed by exposure to 120 dB for final startle. ***U***, Basal startle response in JCNO-treated CaMKIIα-tTA::TRE-hM4Di bigenic male mice was unaltered compared with vehicle-treated CaMKIIα-tTA::TRE-hM4Di bigenic male controls. ***V***, CaMKIIα-tTA::TRE-hM4Di bigenic male mice with a history of JCNO treatment did not show any change in PPI compared with the vehicle-treated controls (*n* = 10 for vehicle; *n* = 9 for JCNO male mice). Results are expressed as the mean ± SEM, and groups are compared using the two-tailed, unpaired Student’s *t* test.

We examined whether chronic CNO-mediated hM4Di-DREADD inhibition of CaMKIIα-positive forebrain excitatory neurons during the juvenile window (P28 to P40) alters anxiety-like behavior in adulthood. We subjected CaMKIIα-tTA::TRE-hM4Di bigenic adult male mice with a history of JCNO treatment to a battery of behavioral tests commencing 2 months after the cessation of CNO treatment. We examined anxiety-like behavior on the OFT, EPM test, and LD test. JCNO-treated CaMKIIα-tTA::TRE-hM4Di bigenic adult male mice did not exhibit any difference in anxiety-like behavior on these behavioral tasks (Fig. 5*A*). On the OFT, we noted no change in the total distance traveled in the OFT arena (Fig. 5*B*,*C*), as well as in the percentage of time spent in the center (Fig. 5*D*), the percentage of distance traveled in the center (Fig. 5*E*), or in the total number of entries to the center of the OFT arena (Fig. 5*F*). We also observed no significant differences in behavior on the EPM test (Fig. 5*G–M*), with no change noted for the percentage of time spent in closed arms (Fig. 5*H*) or open arms (Fig. 5*I*), as well as the percentage of distance traveled in closed arms (Fig. 5*J*) or open arms (Fig. 5*K*). The total number of entries to both the closed (Fig. 5*L*) and open arms (Fig. 5*M*) was also unchanged across vehicle- and JCNO-treated CaMKIIα-tTA::TRE-hM4Di bigenic adult male mice. Behavioral measures assessed on the LD box test (Fig. 5*N–P*) were also not altered between vehicle- and JCNO-treated CaMKIIα-tTA::TRE-hM4Di bigenic adult male cohorts, with no difference noted for either the percentage of time spent in the light box (Fig. 5*O*) or the number of entries to the light box (Fig. 5*P*). Further, we examined whether CaMKIIα-tTA::TRE-hM4Di bigenic adult male mice with a history of JCNO treatment differed from their vehicle-treated controls on the FST (Fig. 5*Q–S*) and TST. JCNO-treated CaMKIIα-tTA::TRE-hM4Di bigenic adult males did not show any significant differences in the percentage of immobility time (Fig. 5*R*) or the number of immobility events (Fig. 5*S*) on the FST. We also noted no significant differences in the percentage of immobility time on the TST between JCNO-treated CaMKIIα-tTA::TRE-hM4Di bigenic adult male and the vehicle-treated cohort (percent of immobility time: vehicle-treated CaMKIIα-tTA::TRE-hM4Di bigenic adult male mice = 64.15 ± 5.56, *n* = 9; JCNO-treated CaMKIIα-tTA::TRE-hM4Di bigenic adult male mice = 60.52 ± 2.32, *n* = 9; results are expressed as the mean ± SEM). Collectively, these results indicate that chronic hM4Di-DREADD-mediated chemogenetic inhibition of CaMKIIα-positive forebrain excitatory neurons during either the early postnatal or juvenile window does not alter anxiety- or despair-like behavior in adulthood.

We next sought to address whether chronic CNO-mediated hM4Di inhibition of CaMKIIα-positive forebrain excitatory neurons in the juvenile window resulted in any change in sensorimotor gating behavior in adulthood. To measure changes in sensorimotor gating, we subjected JCNO-treated CaMKIIα-tTA::TRE-hM4Di bigenic male mice and their respective vehicle-treated control groups to the PPI paradigm (Fig. 5*T*). We noted no significant difference in basal acoustic startle (Fig. 5*U*) or the percentage of prepulse inhibition (Fig. 5*V*) at all prepulse tones tested above the background noise in the JCNO-treated CaMKIIα-tTA::TRE-hM4Di bigenic male mice and their respective vehicle-treated controls. These findings indicate that chronic CNO-mediated hM4Di-DREADD inhibition of CaMKIIα-positive forebrain excitatory neurons during the juvenile window does not result in any significant change in sensorimotor gating.

## Discussion

Our findings indicate that chronic hM4Di-DREADD-mediated inhibition of CaMKIIα-positive forebrain excitatory neurons during the early postnatal or juvenile windows of life does not appear to influence the development of anxiety- or despair-like behavior or alter sensorimotor gating in adulthood. Preclinical studies using rodent models indicate that the first 2 weeks of life represent a critical period window (Baram et al., 1997), wherein the early stress of maternal separation (MS; Benekareddy et al., 2011; Kalinichev et al., 2002; De Melo et al., 2018; Teissier et al., 2020) or pharmacological perturbations that elevate serotonin levels, such as postnatal selective serotonin reuptake inhibitor (SSRI) administration (Ansorge et al., 2004; Ko et al., 2014; Rebello et al., 2014; Sarkar et al., 2014; Soiza-Reilly et al., 2019), can result in the life-long programming of persistent mood-related behavioral changes. Converging evidence across diverse models of early stress has implicated perturbations in G-protein-coupled receptor (GPCR) signaling during these critical periods in the establishment and eventual emergence of disrupted anxiodepressive behaviors (Vinkers et al., 2010; Benekareddy et al., 2011; Sarkar et al., 2014; Teissier et al., 2015; Soiza-Reilly et al., 2019; Pati et al., 2020). This has led to a hypothesis that a balance between G_q_- and G_i_-mediated GPCR signaling within neocortical brain regions during these early developmental windows may be important to shaping the development of trait anxiety and behavioral despair (Lambe et al., 2011; Tiwari et al., 2021). A recent study has shown that enhanced G_q_ signaling via chemogenetic hM3Dq-DREADD-mediated activation of CaMKIIα-positive forebrain excitatory neurons during the postnatal, but not the juvenile or adult, temporal windows results in long-lasting increases in anxiety- and despair-like behavior, accompanied by perturbed sensorimotor gating and PPI deficits (Pati et al., 2020). While several studies have used pharmacological or genetic perturbation studies to examine the contribution of G_i_-coupled GPCRs, in particular the 5-HT_1A_ receptor (Gross et al., 2002; Vinkers et al., 2010; Richardson-Jones et al., 2010, 2011; Garcia-Garcia et al., 2014; Sarkar et al., 2014) during postnatal life in programming mood-related behavior, this has not been directly tested using a chemogenetic-based approach to perturb G_i_ signaling in CaMKIIα-positive forebrain excitatory neurons. Based on prior evidence that pharmacological blockade (Vinkers et al., 2010; Sarkar et al., 2014) or genetic loss of function of the G_i_-coupled 5-HT_1A_ receptor results in enhanced anxiety- and despair-like behavior (Gross et al., 2002; Richardson-Jones et al., 2010, 2011), a working hypothesis would suggest the possibility that enhancing G_i_ signaling in CaMKIIα-positive forebrain excitatory neurons during the postnatal window might evoke a decline in anxiety- and despair-like behaviors in adulthood. Prior evidence indicates that transient hM4Di-DREADD inhibition of the amygdala in infant rhesus monkeys has long-lasting effects on emotionality, with a decline noted in fear and anxiety responses (Raper et al., 2019). A study also shows that constitutive overexpression of the G_i_-coupled 5-HT_1A_ receptors (Kusserow et al., 2004) can program decreased anxiety-like behavior in adulthood. This differs from the pharmacological studies, wherein 5-HT_1A_ receptor stimulation in the postnatal window using the agonist 8-OH-DPAT alone does not modulate anxiety-like behavior, but can increase despair-like behavior in adulthood (Ishikawa and Shiga, 2017), whereas blockade of 5-HT_1A_ receptors in early postnatal life with the selective antagonist WAY 100635 evokes increased anxiety-like behavior (Vinkers et al., 2010; Sarkar et al., 2014). However, there is a paucity of literature that addresses directly whether broad alteration of G_i_ signaling within forebrain neurocircuits during the postnatal temporal window contributes to the programming of altered mood-related behaviors. The results of the present study clearly demonstrate that G_i_-mediated inhibition of forebrain excitatory neurons using the hM4Di-DREADD during either the postnatal (P2 to P14) or juvenile (P28 to P40) windows does not evoke any significant behavioral change on conflict-based tasks assessing anxiety-like behavior, namely the OFT, EPM test, and LD avoidance test in adulthood. Further, we also noted no change in despair-like behavior on the TST and FST or in sensorimotor gating behavior on the PPI in adulthood.

The expression of the hM4Di-DREADD was restricted to the neocortex and hippocampus, as indicated by both Western blotting and immunofluorescence analysis, and the hM4Di-DREADD expression was restricted to CaMKIIα-positive forebrain excitatory neurons and was not observed in PV-positive inhibitory interneurons or GFAP-positive astrocytes (Fig. 1). Western blotting analysis for the neuronal activity marker c-Fos indicates that, as reported previously (Vetere et al., 2017; Salvi et al., 2019), treatment with the DREADD ligand CNO results in reduced neuronal activity in the forebrain of CaMKIIα-tTA::TRE-hM4Di bigenic mice, as indicated by a decline in c-*fos* protein levels. However, one of the caveats of using a Western blotting approach to examine total c-Fos protein levels within the neocortex and hippocampus is that one loses the information of the specific classes of cells exhibiting a c-Fos reduction. While our biochemical studies do indicate a reduction in total c-Fos protein level on CNO-mediated hM4Di DREADD stimulation, further electrophysiological and immunofluorescence studies would aid in uncovering the precise cell types targeted in the neocortex and hippocampus. We found that the administration of the exogenous DREADD ligand CNO in the postnatal or juvenile window did not appear to alter the growth and development of animals, based on observations of no weight change in animals, as well as a normal ontogenic development of reflex behaviors in CNO-treated CaMKIIα-tTA::TRE-hM4Di bigenic rat pups. This suggests that enhanced hM4Di-DREADD-mediated inhibition of CaMKIIα-positive forebrain excitatory neurons in postnatal life does not appear to influence the emergence of critical reflexes such as air-righting, negative geotaxis, and surface righting. This is in agreement with prior studies that indicate that the DREADD agonist CNO during the postnatal window does not appear to influence the emergence of key developmental milestones (Pati et al., 2020).

A change in excitation-inhibition balance during critical developmental time windows, with a shift toward enhanced excitation of forebrain pyramidal neurons and a commensurate reduction in inhibitory tone, has been posited to play a crucial role in the programming of life-long perturbations of mood-related behaviors in several neurodevelopmental disorder models (Yizhar et al., 2011; Fenton, 2015; Nelson and Valakh, 2015; Tatti et al., 2017; Sohal and Rubenstein, 2019). Indeed, hyperexcitation of Emx1-positive neurons from P4 to P14 in the neocortex using either a noninvasive bioluminescent chemogenetics approach (Medendorp et al., 2021) or hM3Dq-DREADD-mediated activation of CaMKIIα-positive forebrain excitatory neurons from P2 to P14 (Pati et al., 2020) resulted in enhanced anxiety-like behaviors and perturbed social behavior. An important experimental counterpart would be to increase inhibition in forebrain pyramidal neurons in these developmental windows and to address the influence on the emergence of mood-related behaviors. Here we provide evidence of the consequences of enhanced hM4Di-mediated DREADD inhibition of forebrain excitatory neurons on the emergence of mood-related behavior and indicate that this perturbation does not appear to influence the development of either anxiety- or despair-like behaviors in both male and female mice. However, we report clear sex differences on specific measures of anxiety- and despair-like behavioral tasks, in agreement with prior studies (Scholl et al., 2019), with enhanced locomotion noted in the OFT in females, accompanied by greater anxiety-like behavior on the OFT, EPM test, and LD box test in the female cohorts. We also find that, despite a clear decline in the percentage of time spent in open arms of the EPM in the vehicle and PNCNO female groups, the number of entries to the open arms were significantly higher, as previously reported (Knight et al., 2021). Our focus was to address whether chronic hM4Di-DREADD-mediated inhibition of CaMKIIα-positive forebrain excitatory neurons during the early postnatal window alters anxiety-like behavior, and our studies indicate no significant effects of PNCNO administration despite the baseline sex differences noted in anxiety- and despair-like behavior uncovered in male and female cohorts. A prior study using viral-based targeting strategies to evoke hM4Di-mediated DREADD inhibition of neurons within the medial prefrontal cortex (PFC) during the postnatal developmental window indicated that this perturbation enhanced anxiety- and despair-like behavior in adulthood, whereas hM3Dq-mediated DREADD activation overlapping with the stress of MS ameliorated the behavioral consequences of early stress in male mice (Teissier et al., 2020). Further, a recent report also examined the influence of chemogenetic inhibition of PFC neurons, which are transiently positive for the serotonin transporter in postnatal life, and showed that the enhanced anxiodepressive behaviors noted in adult animals with a history of postnatal SSRI exposure was exacerbated on hM4Di-DREADD-mediated inhibition of this subclass of PFC neurons in adulthood in both male and female mice. In contrast, hM3Dq-DREADD-mediated activation of these PFC neurons in adulthood ameliorated the postnatal SSRI-evoked anxiety- and despair-like behaviors (Soiza-Reilly et al., 2019). Studies conducted in mice targeting PFC glutamate projection neurons using the CaMKIIα promoter to drive hM4Di-DREADD virally during postnatal life, followed by an acute treatment with CNO in adulthood, indicated no behavioral changes in the FST, OFT, and novelty-suppressed feeding test baseline, but served to exacerbate the anxiodepressive behavioral phenotypes in mice with a history of postnatal fluoxetine treatment (Soiza-Reilly et al., 2019). It is of importance to note that the promoters used in these studies in specific cases target both excitatory and inhibitory neurons of the PFC or a subclass of raphe-projecting PFC neurons further the use of transgenic mice versus viral-mediated gene delivery, and differences in the developmental epoch targeted and nature of experimental paradigms make it challenging to directly compare our findings with these studies, in particular given that we targeted hM4Di-mediated DREADD expression to all CaMKIIα-positive forebrain excitatory neurons and these prior studies assessed effects on a subset of PFC neurons.

While we observed no change in anxiety-like behavior on the OFT, EPM test, and LD avoidance test, we did note that PNCNO-treated CaMKIIα-tTA::TRE-hM4Di bigenic male mice exhibited a higher basal acoustic startle response, although they showed no change in PPI behavior. An enhanced baseline acoustic startle response has been suggested to be reflective of enhanced anxiety-like behavior (Grillon, 2008); however, we see no indication of perturbation in anxiety on any of the conflict-anxiety behavioral tasks. We cannot preclude the possibility of a developmental perturbation of acoustic sensory circuits, given the driver we have used is broad based, and the hM4Di-DREADD would be driven in all forebrain excitatory neurons. One of the caveats of our sensorimotor gating studies is that we have restricted our PNCNO experiments to CaMKIIα-tTA::TRE-hM4Di bigenic male mice in adulthood, and so we cannot preclude the possibility that there could be effects on sensorimotor gating in female mice subjected to PNCNO-mediated hM4Di-DREADD inhibition of CaMKIIα-positive forebrain excitatory neurons. Collectively, our results suggest that enhancing G_i_ signaling in forebrain excitatory neurons during the postnatal window does not influence the programming of anxiety- and mood-related behaviors in both male and female mice; however, it is vital to keep in mind that this is not the same as driving enhanced G_i_ signaling via a specific GPCR, such as the 5-HT_1A_ receptor, and indeed it is possible that a more targeted approach to selectively enhance 5-HT_1A_ receptor signaling in forebrain excitatory neurons in this developmental window could exert a role in programming changes in emotionality. It would also be of interest to address whether the hM4Di-DREADD-mediated inhibition of forebrain excitatory neurons in this critical temporal window can influence performance on cognitive tasks, which has not been addressed in the present study.

In our study, we have also addressed whether hM4Di-DREADD-mediated inhibition of forebrain excitatory neurons in another critical temporal window implicated in shaping mood-related behavioral traits, namely the juvenile window, could impact anxiety- and despair-like behaviors (Brydges et al., 2012, 2014; Hollis et al., 2013; Luo et al., 2014; Suri et al., 2015; Albrecht et al., 2017). Animals subjected to stress during the juvenile window exhibit increased anxiety-like behavior, show enhanced benzodiazepine sensitivity, and can establish a heightened vulnerability to adult-onset stress (Avital and Richter-Levin, 2004). Peripubertal stress also causes alterations in GABAergic neurotransmission (Tzanoulinou et al., 2014), and GAD65 haplodeficiency has been shown to be associated with resilience to juvenile stress-induced increased anxiety-like behavior, possibly because of delayed maturation of inhibitory signaling (Müller et al., 2014). Previous studies have shown that enhanced G_q_-mediated signaling via a chemogenetic approach in the juvenile window does not influence anxiety-, despair-, or schizophrenia-like behavior (Pati et al., 2020). The present work indicated that hM4Di-DREADD inhibition of forebrain excitatory neurons during the juvenile temporal window does not appear to alter anxiety- or despair-like behaviors and does not influence sensorimotor gating in male mice. One of the lacunae of our study is that we restricted all juvenile perturbations to male mice and hence cannot comment on whether hM4Di-DREADD inhibition of forebrain excitatory neurons during the juvenile temporal window modulates anxiodepressive behaviors and sensorimotor gating in female mice.

The profiling of the behavioral consequences of hM4Di-DREADD inhibition of forebrain excitatory neurons in postnatal or juvenile life suggests that this perturbation does not alter the emergence of anxiety, despair, and sensorimotor gating behavior on a variety of behavioral tasks in adulthood. This differs quite starkly from our recent study wherein hM3Dq-DREADD-mediated activation of forebrain excitatory neurons in postnatal, but not juvenile or adult, life resulted in persistent increases in anxiety-, despair-, and schizophrenia-like behavior, accompanied by specific molecular, metabolic, and functional changes in the both the neocortex and the hippocampus (Pati et al., 2020). While we do not observe any change in mood-related behaviors following hM4Di-DREADD inhibition of forebrain excitatory neurons, we cannot preclude the possibility that these animals may exhibit differential responses to a stressor experience in adulthood. Our work motivates future investigation to address in detail how perturbations in GPCR signaling within forebrain circuits during critical developmental time windows may shape vulnerability or resilience to adult-onset stressors.

## Synthesis

Reviewing Editor: Nathalie Ginovart, Universite de Geneve Decisions are customarily a result of the Reviewing Editor and the peer reviewers coming together and discussing their recommendations until a consensus is reached. When revisions are invited, a fact-based synthesis statement explaining their decision and outlining what is needed to prepare a revision will be listed below. The following reviewer(s) agreed to reveal their identity: Mariano Soiza-Reilly.

Upon consultation with two reviewers, we have reached the decision of “Revise and Re-review” on your manuscript investigating the effects of early life inhibition of forebrain glutamate neurons on mood-related behavior in adulthood. Both reviewers found interest in the data presented but also raised some concerns regarding the manuscript as currently written and agreed that these revisions should require less than 2 months’ work. In particular, there was a clear agreement that complete datasets including both males and females should be presented and that the statistics should be revised to use two-way ANOVAs rather that Student’s t-tests, considering “sex” as an independent variable in the analyses. Both reviewers also raised some additional issues, that should also be addressed in the revision. For instance, the authors should better explain their choice of the two different PN periods and for letting out the P14-P28 period in their investigation. Further details should also be added in the methods as a lack of detail in the procedures makes interpretation of the data challenging. Also, extended data could be included into the main figures, for instance data pertaining to locomotor activity... Please find the specific comments and queries raised by the reviewers below:

Reviewer 1 :

The manuscript “Chronic hM4Di-DREADD mediated chemogenetic inhibition of forebrain excitatory neurons in postnatal or juvenile life does not alter adult mood-related behavior” (eN-NRS-0381-21) investigates the effects of in vivo chemogenetic inhibition of forebrain glutamate neurons during different developmental periods of high relevance for mood and stress-related psychiatric disorders. The study is fairly written and I have few comments/suggestions to the authors that could help to improve the manuscript.

Comments:

- Authors performed controls of inhibition 30 min after a single CNO exposure at each of the studied periods by western blot. However, I wonder if they have tried to visualize local DREADD activation by c-Fos/CamKII co-immunostainings? If possible, this would be perhaps more accurate and will help to understand the apparent lack of c-Fos inhibition shown in the hippocampus after CNO exposure at P35.

- Regarding Figure 1, could you increase the size of labels within panels? Also, CamKII-HA co-labeling in Panel A is not very obvious. Please improve the images and use arrows to show double-labeled cells. In Panel C, CamKII staining in the hippocampus is surprisingly low, please add a more representative image. Something similar happens in the cortex (Panel D), please improve images. Regarding the cortex, have you seen any layer-specific preferential recombination in your mice? In Panel J, the upper blot does not seem to be quite representative of a c-Fos inhibition in the hippocampus.

- Data from Figures 3 and 4 should be analyzed together considering “Sex” as a factor in a two-way ANOVA (in addition to the “Treatment” factor).

- Data from females exposed to CNO at the later period (P28-40) are missing. This should be added to make possible sex comparisons as before. The same comment for despair-like behavioral data. Have you tried to analyze immobility time min by min in a sort of time course? Sometimes this kind of analysis helps to understand the behavioral response.

- Female data is missing in the startle response experiments. In general, if female data are not added to the study, then all female data should be removed, and in the title it should be clarified that the study was done in males.

- In the first paragraph of the Discussion, I think authors wanted to cite as a reference to Teissier et al 2019 instead of 2015.

- In the Discussion, how do you fit your hypothesis of anxiety and despair-like behavioral declining with the enhancement of Gi-signaling with previous data showing that in the SERT-KO, an enhancement of 5-HT1A signaling in the PFC is thought to mediate the long-lasting increases in anxiety and despair-like behaviors?

- In the Discussion, when mentioning the promoter used in the study by Soiza-Reilly et al. 2019, please consider that in part that study also uses CamKII promoter to drive hM4D expression in the PFC.

Reviewer 2 :

This manuscript investigates the effects of chronic DREADD-based enhanced Gi signaling throughout forebrain neurons on adult anxiety-like and despair-like behavior. The authors find that found that chronic administration of CNO to activate genetically encoded hM4Di in biogenic mice during postnatal lie (p 2-P 14) or during the juvenile or peri-adolescent person (P 28-P40) had no effect on body weigh, and the normal ontogeny of reflex behaviors in pups as well as no detectible effect on anxiety-like and despair-like behavior in adulthood.

The rationale for the manipulation is not quite clear to this reviewer. The authors cite data suggesting the increased Gi signaling in forebrain circuits is associated with resilience and thus it not quite clear what the authors expected to observe in the standard battery of anxiety-like behavior tasks. Details of the lighting conditions are not presented and thus it is not clear if the environmental conditions during testing were simply not aversive enough to detect a difference in behavior during adulthood. One would expect some differences in the forced swim test, but the authors could consider adding a stress challenge to determine if enhance Gi signaling promotes resilience. In addition, given the level of hippocampal expression in these mice and the use of it in verification, it is surprising that a hippocampal-dependent task was not also chosen as part of the behavioral assay.

The authors conducted most behavior on males and females but sex was not run as a variable in the statistical analysis, nor were the results presented in a way to directly compare sex. Although there were no differences in treatment groups between sex, it would be advisable to compare sex and show any differences in the behaviors (it appears as if there are some). This would require running ANOVA’s rather than t-tests.

For juvenile experiment there seems to be only male data presented? What is the reasoning for this? This applies to data presented in all of Figure 5 and Figure 6G. The same applies for the PPI experiment in which only male mice were used.

Could the authors clarify why P14-P27 was left out as a time period for administration of CNO? This is likely to produce a separation in treatments between the two distinct ages, but it is not explicitly stated in the text.

## Author Response

Response to reviewers:

1. Authors performed controls of inhibition 30 min after a single CNO exposure at each of the studied periods by western blot. However, I wonder if they have tried to visualize local DREADD activation by c-Fos/CamKII co-immunostainings? If possible, this would be perhaps more accurate and will help to understand the apparent lack of c-Fos inhibition shown in the hippocampus after CNO exposure at P35.

We appreciate the reviewer’s point that looking at c-fos/CaMKII co-immunofluorescence might better indicate a decline in the c-Fos activity in the CamKII-alpha forebrain neurons. However, for this, the tissue processing requires that we do this with perfused vehicle and CNO-treated CamKIIα-tTA::TRE-hM4Di bigenic brains, which we do not have access to at present. We had harvested the tissues for western blotting, because we thought that it would be a more quantitative approach. We cannot preclude the possibility that we might have missed catching a c-Fos inhibition given that we are looking at the total c-Fos protein levels in the entire hippocampal tissue, which might mean that even if there is a decline in c-Fos activity in CaMKII-positive subset of neurons, we may not be able to catch it. We do see a clear decline in c-Fos levels in the postnatal window in both the hippocampus and cortex, and in the cortex in the juvenile window but do not detect it in the hippocampus in the juvenile window. To repeat these experiments again using co-immunofluorescence we would have to set up an entire new set of bigenic breedings to harvest tissues at P7 and P35, which requires substantial animal breeding and was not possible in the time period granted to us for revision as we have substantially scaled back the colony. However, we do agree with the reviewer that this is an important point, and we may have missed a decline in c-Fos expression selectively in the CaMKII-positive neurons, given that we had used a more quantitative western blotting approach which loses information of cell-type specificity. We have now specifically mentioned this caveat in the discussion section to point some of the lacunae with the approaches we have used.

2. Regarding Figure 1, could you increase the size of labels within panels? Also, CamKII-HA co-labeling in Panel A is not very obvious. Please improve the images and use arrows to show double-labeled cells. In Panel C, CamKII staining in the hippocampus is surprisingly low, please add a more representative image. Something similar happens in the cortex (Panel D), please improve images. Regarding the cortex, have you seen any layer-specific preferential recombination in your mice? In Panel J, the upper blot does not seem to be quite representative of a c-Fos inhibition in the hippocampus.

We have provided better representative images (see modified Figure 1) as requested by the reviewers by redoing the immunofluorescence in another set of CamKIIα-tTA::TRE-hM4Di (n = 5). We do not see any layer specific preferential recombination in our animals as revealed by the HA staining. With regards to the reviewers’ suggestion to include a better representative c-Fos western blot image for c-Fos in the hippocampus, we have provided a lower exposure to avoid any saturation that might make it difficult to note the decline. We had normalized c-Fos to actin as a loading control for the experiment and across an (n = 5/group) we do note a significant decline.

3. Data from Figures 3 and 4 should be analyzed together considering “Sex” as a factor in a two-way ANOVA (in addition to the “Treatment” factor).

As suggested by the reviewer, we have now reanalyzed al the PNCNO data from OFT, EPM, LD Box and FST which was performed on both male and female mice via a two-way ANOVA analysis considering PNCNO treatment and Sex as the two variables. We also have modified our Figure 3, and Figure 4 and our statistical table to reflect this new analysis. We also do note strong main effects of Sex for multiple behavioral measure on tasks for anxiety and despair-like behavior. We have changed our results section to reflect this analysis and modified discussion to also comment appropriately. We appreciate the reviewers suggestion to look at our data with the variable of sex as a factor, and while we dont really see any substantial PNCNO x Sex interaction, or significant main effects of PNCNO, we do observe significant main effects of Sex on multiple measures.

4. Data from females exposed to CNO at the later period (P28-40) are missing. This should be added to make possible sex comparisons as before. The same comment for despair-like behavioral data. Have you tried to analyze immobility time min by min in a sort of time course? Sometimes this kind of analysis helps to understand the behavioral response.

We appreciate the point raised by the reviewers that data from females exposed to juvenile CNO treatment would be a valuable addition to the manuscript. When we originally designed the experiments we first focussed on the PNCNO treatments and ran them for both male and female mice. These are large experiments with substantial breeding cohorts to get the numbers required to run the various different tests, particularly as we try to derive animals from distinct litters to avoid any litter-specific effects. When we noted absolutely no effect of CNO in the PNCNO experiment, we decided to analyse the juvenile CNO cohort with the males to assess if we noted any changes in anxio-depressive behaviors. We did not observe any change on multiple measures post JCNO treatment in male bigenic mice. We agree that a female cohort would be immensely valuable and we really believe that this is a lacunae in our study. We have very clearly stated that this is a lacunae and we agree that we should run the experiment in both males and females. However, at this juncture to set up an entire new breeding cohort to get juvenile CamKIIα-tTA::TRE-hM4Di bigenic female mice and then assess their behavior in adulthood was not possible given the timelines for revision. If the reviewers feel that it is best we drop the entire juvenile data we are willing to do so, although what we have done at this stage is to clearfly caveat our experiments, state that it is critical we address effects in females and tone down the conclusions we are drawing in the absence of a female JCNO cohort. We believe that the results from the JCNO male mice indicate that at least in males there is no effect of this treatment on anxio-depressive behaviors and sensorimotor gating, but we do state that we cannot preclude possible effects on female mice as we have not performed this particular experiment.

As suggested by the reviewer we did break down our FST analysis into time bins and we find that this does not change the results and so we have included the cumulative data in the figures. We have included below for the reviewers the time-binned analysis indicating no effect of PNCNO in males and females and JCNO in males on the FST.

Shown above is the analysis for FST after breaking down the data into one-minute time bins from 0 to 5 minutes. We do not note a change in any time bin for percent immobility time between vehicle and PNCNO-treated males (A), females (C) , as well as between vehicle and JCNO-treated males (E). Number of immobility events also remained unchanged across all time bins for PNCNO-treated males (B), females (D) , as well as JCNO-treated males (F) when compared with their vehicle-treated controls.

5. Female data is missing in the startle response experiments. In general, if female data are not added to the study, then all female data should be removed, and in the title it should be clarified that the study was done in males.

As stated in our response to point 4 from the reviewers we agree that this is a caveat to our manuscript. We have carried out OFT, EPM, LD Box and FST from the PNCNO experiment in both males and females. We have examined sensorimotor gating in PNCNO males and the JCNO experiments are all done in male mice. We realise that an ideal design would have been examining all readouts in both male and female mice for PNCNO and JCNO. However, the scale of breeding required to generate bigenic CamKIIα-tTA::TRE-hM4Di mice which are initially maintained back-crossed to C57BL6 mice and double positives cohorts are generated only for breeding for experimental purposes, as well as to ensure we derive animals across litters to avoid any litter-specific effects, the number of animals to be used was fairly large and so we restricted ourselves to the male mice for the JCNO cohorts and for the sensorimotor gating experiments. We completely agree with the reviewers that as a consequence we are missing some equivalent data from female mice in the JCNO cohorts and for PNCNO startle behavior. We have very clearly discussed that this is a lacunae in our data and upfront highlighted the issues with this in our discussion. This manuscript is submitted under the negative results category of eNeuro and we feel that even the absence of effects in JCNO male mice may be worthwhile to include despite the lack of a comparative female cohort especially if we state the lacunae clearly. We appeal to the reviewers to kindly consider this request. However, if the reviewers do feel we should remove all female data we would rather take out the JCNO male experiment and restrict our manuscript to only PNCNO experiments.

6. In the first paragraph of the Discussion, I think the authors wanted to cite as a reference to Teissier et al 2019 instead of 2015.

We thank the reviewer for pointing out this oversight. We have now changed the reference.

7. In the Discussion, how do you fit your hypothesis of anxiety and despair-like behavioral declining with the enhancement of Gi-signaling with previous data showing that in the SERT-KO, an enhancement of 5-HT1A signaling in the PFC is thought to mediate the long-lasting increases in anxiety and despair-like behaviors?

We thank the reviewers for raising this point. There are several reports that show that absence of 5-HT1A signalling, which is Gi-coupled, either by pharmacological intervention using an antagonist in early life (Vinkers et al, 2010; Sarkar et al., 2014) or by using a genetic knockout model (Gross et al 2002; Ramboz et al, 1998), results in increased anxio-depressive behavior. The SERT-KO studies show an enhanced anxiety and despair-like phenotype ;likely due to elevated developmental exposure to heightened serotonin levels. It is noteworthy to consider that the serotonin receptor milieu in a SERT-KO is likely to be quite distinct given the heightened levels of serotonin across life. In this context, enhanced PFC 5-HT1A receptor signalling may contribute to the enhanced anxiety in the SERT KO genotype, but may not play that role in the wild-type background. Indeed, the idea that driving enhanced Gi signaling in a subset of raphe-projecting serotonin neurons can exacerbate the postnatal fluoxetine evoked anxiety in adulthood, suggests that enhanced 5-HT1A signaling in perhaps a subset of PFC neurons may be vital to the persistent anxiodepressive behaviors that arise after postnatal elevation of serotonin. It is difficult to directly compare this to our work as the bigenic line we use would target all CamKIIα forebrain excitatory neurons and that might not reproduce what is seen with a far more targeted approach to enhance5-HT1A signaling in a subset of PFC neurons, most of them likely project to the raphe. We have added some components of this to our discussion section to highlight the differences.

8. In the Discussion, when mentioning the promoter used in the study by Soiza-Reilly et al. 2019, please consider that in part that study also uses CamKII promoter to drive hM4D expression in the PFC.

We thank the reviewer for this point and we have now changed our discussion to reflect this study.

9. The rationale for the manipulation is not quite clear to this reviewer. The authors cite data suggesting the increased Gi signaling in forebrain circuits is associated with resilience and thus it is not quite clear what the authors expected to observe in the standard battery of anxiety-like behavior tasks. Details of the lighting conditions are not presented and thus it is not clear if the environmental conditions during testing were simply not aversive enough to detect a difference in behavior during adulthood. One would expect some differences in the forced swim test, but the authors could consider adding a stress challenge to determine if enhanced Gi signaling promotes resilience. In addition, given the level of hippocampal expression in these mice and the use of it in verification, it is surprising that a hippocampal-dependent task was not also chosen as part of the behavioral assay.

Our rationale for this experiment described in our introduction is based on (1) pharmacological and genetic studies modulating the Gi-coupled 5-HT1A receptor, wherein loss of function or blockade in the postnatal window results in persistent increases in anxiety and despair-like behavior; and (2) recent work (Pati et al., 2020) that indicates a long-lasting increase in anxio-depressive behavioral phenotypes following hM3Dq-DREADD mediated activation of forebrain excitatory neurons in postnatal life. We hypothesized that enhanced Gi-signaling and DREADD inhibition in forebrain excitatory neurons during postnatal life might evoke a decline in anxio-depressive behaviors, given DREADD activation of these neurons drives robust increases in anxio-depressive behaviors and perturbs sensorimotor gating. However, we did not find any change in behavior on the OFT, EPM, LD box, FST in male and female mice in the PNCNO cohort. We clearly see strong sex differences on behavior in some of these tasks, as has been reported previously leading us to believe that the tests are robust and would be likely to pick up differences should they exist. All our conflict-anxiety paradigms are performed in the dark, except the light-dark avoidance test, which was done in a standard lit room. We did put these animals through a social isolation challenge but did not observe any change in behavior (data not included). We agree that it would be useful to add in some hippocampal-dependent behaviors, however we have not performed cognitive tasks in this cohort and this is now indicated in the discussion.

10. The authors conducted most behavior on males and females but sex was not run as a variable in the statistical analysis, nor were the results presented in a way to directly compare sex. Although there were no differences in treatment groups between sex, it would be advisable to compare sex and show any differences in the behaviors (it appears as if there are some). This would require running ANOVA rather than t-tests.

We have now reanalyzed our data with a two-way ANOVA using sex as a variable along with PNCNO treatment.

11. For juvenile experiments there seems to be only male data presented? What is the reasoning for this? This applies to data presented in all of Figure 5 and Figure 6G. The same applies for the PPI experiment in which only male mice were used.

We have responded to this specific point in our response to query number 4 and 5.

12. Could the authors clarify why P14-P27 was left out as a time period for administration of CNO? This is likely to produce a separation in treatments between the two distinct ages, but it is not explicitly stated in the text.

For the PNCNO treatment we followed the treatment windows used for many early models (maternal separation: P2-14: elevation of serotonin with postnatal fluoxetine: critical window P2-11), for JCNO we used the juvenile window P28-P40 a post-weaning window. We agree with the reviewers that we have not covered all the early windows, namely P14-P27 and we have now stated this in our methods section. The goal was to test in two distinct windows of postnatal life and juvenile life, however a study that includes an interim window would also be useful
